# Supplementary figures and images for: Histone H4 potentiates neutrophil inflammatory responses to influenza A virus: Down-modulation by H4 binding to C-reactive protein and Surfactant protein D
Source: PLoS One. 2021 Feb 26;16(2):e0247605. doi: 10.1371/journal.pone.0247605 (PMC7909658; doi:10.1371/journal.pone.0247605)

**A**

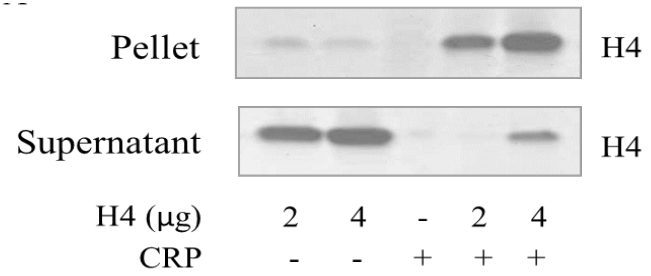

**B**

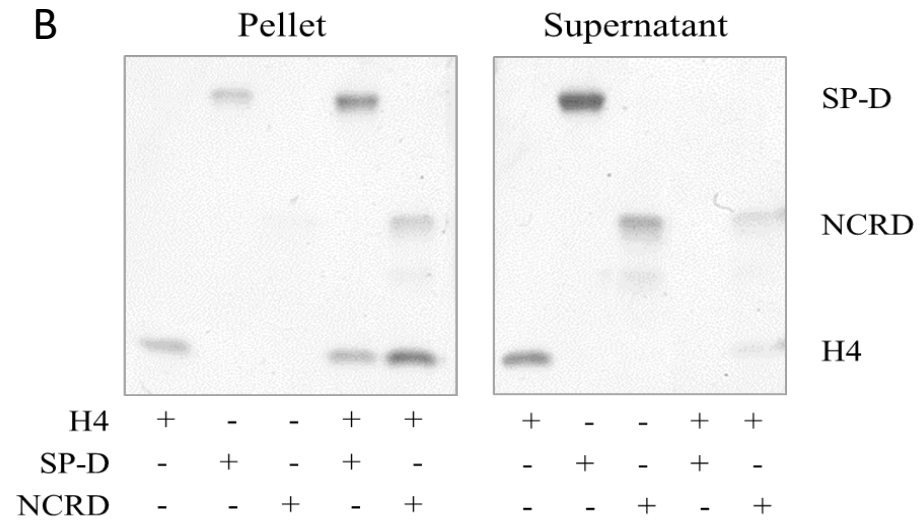

Supplement: S1 File — (PDF) [file pone.0247605.s001.pdf]
